# Effects of Metformin Combined with Lactoferrin on Lipid Accumulation and Metabolism in Mice Fed with High-Fat Diet

**DOI:** 10.3390/nu10111628

**Published:** 2018-11-02

**Authors:** Qing-Qing Min, Li-Qiang Qin, Zhen-Zhen Sun, Wen-Ting Zuo, Lin Zhao, Jia-Ying Xu

**Affiliations:** 1 Department of Nutrition and Food Hygiene, School of Public Health, Soochow University, Suzhou 215123, China; qqmin@stu.suda.edu.cn (Q.-Q.M.); qinliqiang@suda.edu.cn (L.-Q.Q.); Sunny_RainlyMM@163.com (Z.-Z.S.); 2 Jiangsu Key Laboratory of Preventive and Translational Medicine for Geriatric Disease, Soochow University, Suzhou 215123, China; 3 2016 Undergraduate of Clinical Medicine, Soochow University, Suzhou 215123, China; 1630408003@stu.suda.edu.cn; 4 State Key Laboratory of Radiation Medicine and Protection, School of Radiation Medicine and Protection, Soochow University, Suzhou 215123, China; zhaolin.0000@163.com

**Keywords:** metformin, lactoferrin, lipid, liver, obesity

## Abstract

Metformin (Met) and lactoferrin (Lf) both exhibit beneficial effects on body weight management and lipid accumulation. However, the synergistical action of Met and Lf remains unclear. In this study, 64 mice were divided into five groups, namely, the control group, high-fat diet (HFD group), HFD with Met (Met group), Lf (Lf group), and a combination of Met and Lf (Met + Lf group). Met (200 mg/kg body weight) and Lf (2 g/100 mL) were administrated in drinking water. The experiment lasted for 12 weeks. Body weight, serum, and hepatic lipids were determined. Histology of the liver and perirenal fat was observed. Protein expression related to hepatic lipid metabolism was also measured. HFD significantly increased body weight, visceral fat weight, and lipid profiles, which lead to obesity and dyslipidemia in mice. Compared with the HFD group, the treatments significantly decreased body weight and Lee’s index (body mass index of mice) with the lowest values in the Met + Lf group. The treatments also decreased the weight of visceral fat, and improved circulating lipid profile and the ability for regulating glucose intake. The adipocyte size and serum TC level were significantly lower in the Met + Lf group as compared with those in the Met or Lf group. The treatments alleviated hepatic lipid accumulation, especially in the Met + Lf group. For protein expression, the p-AMPK/AMPK ratio, a key kinase-regulating cellular energy homeostasis, was significantly higher in the Met + Lf group than the ratio in the HFD group. Similarly, the treatments significantly downregulated the protein expression of lipogenic enzymes (FAS, ACC, and SREBP-1) and upregulated the protein expression of lipolytic enzyme (ATGL). The protein expression of HMGCoAR, which is an important rate limiting enzyme in cholesterol biosynthesis, was only significantly lower in the Met + Lf group than in the HFD group. In conclusion, Met and Lf, either alone or in combination, prevented HFD-induced obesity and improved lipid metabolism.

## 1. Introduction

Obesity has become a global health problem as it is associated with various complications, such as hyperlipidemia, hypertension, type 2 diabetes mellitus, cardiovascular disorders, and cancer [1,2]. With the prevalence of obesity and its serious related diseases, an urgent need to decrease its growth is present.

Metformin (Met) is widely used as a hypoglycemic agent. Met exhibits beneficial effect on weight loss of obese individuals [3] and western diet-induced mouse models [4]. Met treatment also decreased adipose tissue weight, improved lipid profiles, and lightened the severity of high fat induced hepatic steatosis in human subjects and obese mice [5,6,7,8]. However, Met has transient mild gastrointestinal adverse effects, such as nausea, vomiting, and diarrhea, especially during the initiation of therapy [9]. Therefore, Met is often administered with other drugs and nutrients, such as celecoxib [10], flos lonicera [11], leucine [12], and Vitamin D3 [13], to potentiate the treatment efficacy and reduce the side effects.

Lactoferrin (Lf) is a kind of milk protein that is mainly found in exocrine secretions, such as milk, saliva, and tears [14]. Lf possesses anti-inflammatory, antioxidant, antimicrobial, and immunoregulatory activities [15,16,17,18]. Animal studies have demonstrated that oral Lf administration significantly decreased fat tissue weight, hepatic lipid accumulation, and improved lipid profiles on different diets [19]. A human clinical trial also reported that Lf consumption decreased body weight and visceral fat accumulation in subjects with abdominal obesity [20]. 

Thus, Met and Lf are both effective in lowering circulation lipid level and lipid accumulation. In vivo and in vitro studies also proved that Met and Lf exhibit the ability to activate AMP-activated protein kinase (AMPK), which is a key kinase regulating cellular energy homeostasis [21,22]. Since Lf was considered very safe and has the similar mechanism with Met for the control of obesity, we investigated whether chronic supplementation with Met, Lf, and their combination exerts a direct effect on the improvement of fat accumulation in mice fed with high-fat diet (HFD), and focus on the expression of proteins related to hepatic lipogenesis and lipolysis. 

## 2. Materials and Methods

### 2.1. Animals and Diets

Four-week-old male C57BL/6 mice were obtained from Shanghai Laboratory Animal Company (Shanghai China) and housed under 12 h/12 h light/dark cycle at a constant temperature of 22 ± 2 °C and 60% humidity. After one week of acclimatization, 64 animals were randomly divided into five groups: (1) control group (CON, *n* = 8), (2) HFD group (*n* = 14), (3) HFD plus Met (Met, *n* = 14), (4) HFD plus Lf (Lf, *n* = 14), and (5) HFD plus Met and Lf with the same dosage applied to aforementioned two groups (Met + Lf, *n* = 14). Control diet (10% calories from fat, D12450J) and HFD (60% calories from fat, D12492) were purchased from Research Diets Inc. (New Brunswick, NJ, USA). Met was obtained from Tokyo Chemical Industry Co., Ltd. (Tokyo, Japan). Lf was obtained from Hilmar Cheese Company (Hilmare, CA, USA). According to the literature, Met and Lf were set at 200 mg/kg body weight and 2 g/100 mL, respectively [23,24]. Both were dissolved in distilled water and the drinking water was changed every two days. The animals had access to water and diets at all the times. The experiment lasted for 12 weeks. All the procedures were performed in accordance with the Guidelines in the Care and Use of Animals and with the approval of Soochow University Animal Welfare Committee.

### 2.2. Body Weight, Waist Circumference, and Lee’s Index

During the whole experiment, body weights were measured weekly. After 12 weeks of feeding, the waist circumference and body lengths (nose to anus) were measured [25]. To determine the body mass index of mice, we calculated Lee’s index with the formula [body weight (g) × 1000/body length (cm)]^1/3^.

### 2.3. Oral Glucose Tolerance Test (OGTT)

Before the end of the experiment, blood samples were obtained from a cut on the tail vein after overnight food deprivation. Fasting blood glucose (FBG) was immediately determined using the Roche blood glucose meter (F. Hoffmann-La Roche Ltd., Basel, Switzerland). Then, the mice were given an oral glucose bolus of 2 g/kg. Blood glucose levels were determined at 30, 60, and 120 min. FBG is the level at 0 min. The trapezoidal rule was used to determine the area under the curve (AUC) of the OGTT. 

### 2.4. Sample Collection at Autopsy

With a three-day washout after the OGTT, the mice were deprived of food for 12 h and sacrificed. Blood samples were collected from the retrobulbar vein. Serum was separated by centrifugation and stored at −80 °C. The liver and visceral fat samples (except for mesenteric fat) were dissected and rapidly weighed. Portions of the liver and perirenal fat were fixed immediately in 10% formalin for future histological observation and the remaining portions were stored at −80 °C until for further use.

### 2.5. Serum Biochemical Determination

The serum triglyceride (TG), total cholesterol (TC), HDL-cholesterol (HDL-C), and LDL-cholesterol (LDL-C) were analyzed using commercial enzymatic assay kits (Najing jiancheng Bioengineering Institute, Nanjing, China). Serum levels of leptin and adiponectin were determined using the enzyme-linked immunosorbent assay kit from Cloud-Clone Corp. (Katy, TX, USA) and EMD Millipore Corporation (Darmstadt, Germany). Serum was also used to determine the activities of aspartate transaminases (AST) and alanine transaminase (ALT) by adopting the blood biochemistry analyzer (Ci8200, Abbott Laboratories, Abbott Park, IL, USA).

### 2.6. Hepatic TG and TC Contents

Approximately 10% of the hepatic homogenate was prepared in ice-cold normal saline and centrifuged at 2500× *g* for 10 min. The supernatant was obtained to determine the hepatic TG and TC contents using commercial kit (Najing Jiancheng Bioengineering Institute, Nanjing, China).

### 2.7. Histology of Liver and Perirenal Fat

The formalin-fixed liver and perirenal fat tissues were embedded in paraffin and cut into 6 μm slices. The sections were deparaffinized in xylene and rehydrated by serially washing with decreasing ethanol concentration. The sections were stained using hematoxylin-eosin (H and E) stain and mounted in a xylene-based mounting media. Oil Red O staining in liver tissue was performed on the frozen section of formalin-fixed livers according to the routine procedure [26]. The samples were observed and photograph under a microscope (IX3-AN, Olympus, Japan).

### 2.8. Western Blot Analysis in Liver

Liver tissue samples were homogenized in ice-cold lysis buffer, centrifuged and supernatants were collected. Protein concentration was then measured according to the BCA protein assay kit (Beyotime Institute of Biotechnology, Nantong, China). After denaturation, equal amount of protein (50 μg) were separated by 10% SDS-PAGE and then transferred to a PVDF membrane. The membranes were blocked with 5% skim milk in Tris-buffered saline Tween-20 solution for 1 h and then were incubated overnight with appropriate primary antibodies at 4 °C. The antibodies included phosphorylated(p)-AMPK (1:500, ImmunoWay Biotechnology Company, Plano, TX, USA), AMPK (1:500, ImmunoWay), Sterol regulatory element-binding protein 1 (SREBP-1, 1:500, ImmunoWay), Fatty Acid Synthase (FAS, 1:1000, Cell Signaling Technology, CST, Danvers, MA, USA), Acetyl-CoA Carboxylase (ACC, 1:1000, CST), Adipose triglyceride lipase (ATGL, 1:1000, CST), 3-hydroxy-3-methylglutaryl-Coenzyme A reductase (HMGCoAR, 1:1000, Abcam, Cambridge, UK) and β-actin (1:1000, EMD Millipore, Darmstadt, Germany). After being washed three times in TBST and incubated with appropriate secondary antibodies. Proteins were detected using chemiluminescene ECL Detection Systems (Millipore) and the band intensities were quantified using Image J software. 

### 2.9. Statistical Analysis

All data are expressed as the mean ± standard error (SE). We tested the differences between the groups by one-way analysis of variance (ANOVA, SPSS21) followed by the LSD post hoc test. Difference were considered statistically significant when *p* < 0.05.

## 3. Results

### 3.1. Body Weight, Waist Circumference, and Lee’s Index

**Figure 1:**
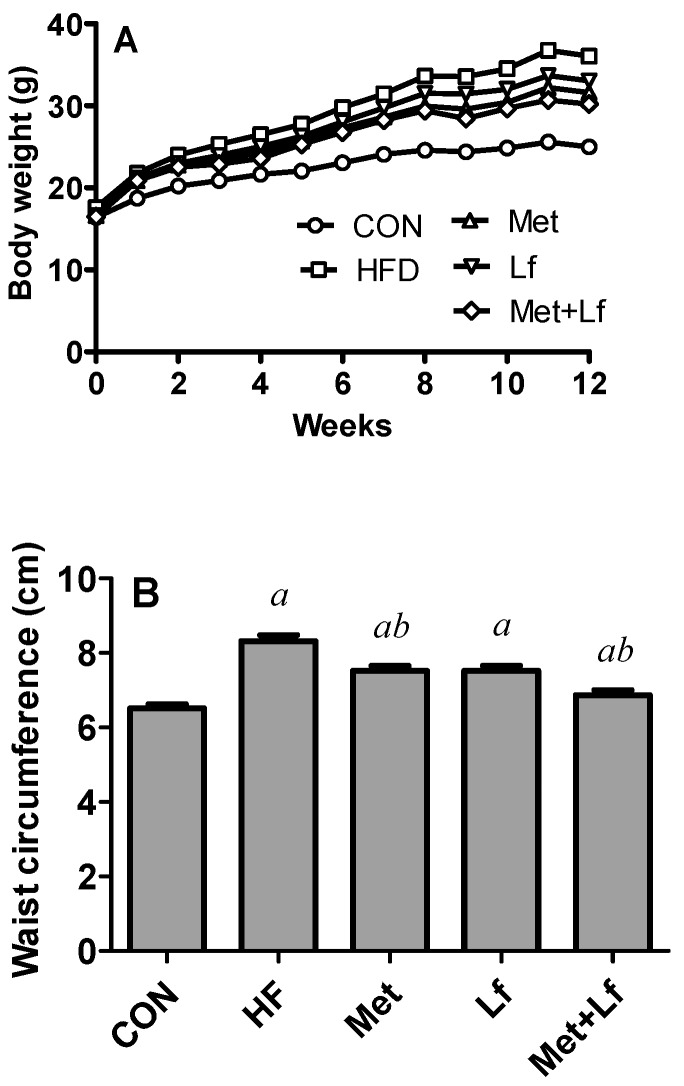

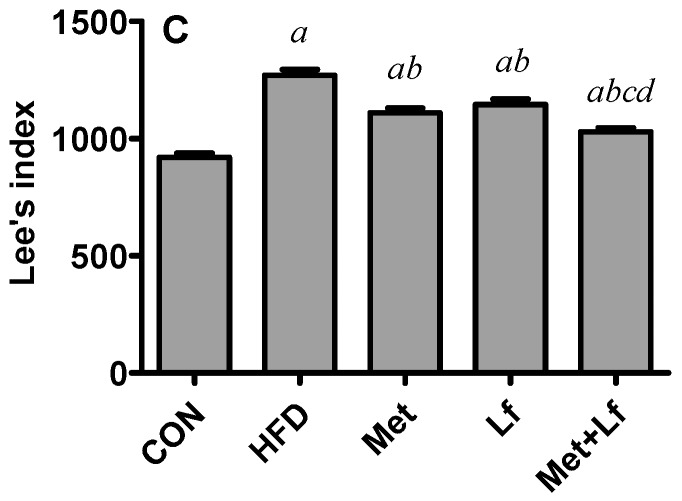
Effect of Met and Lf on body weight (**A**), waist circumference (**B**), and Lee’s index (**C**) in the five groups. Values are express as mean ± SE. ^a^ *p* < 0.05 from CON group; ^b^ *p* < 0.05 from HFD group; ^c^ *p* < 0.05 from Met group; ^d^ *p* < 0.05 from Lf group; CON: control group; HFD: high-fat diet; Met: Metformin; Lf: lactoferrin.

Body weights among the five groups (approximately 16.8 g) were comparable at baseline and increased during experiment. Body weight was significantly heavier in the HFD group than in the control group after three weeks. At the end of the experiment, treatment with Met, Lf, and the combination of the two significantly decreased body weight compared with the HFD group. The body weight in the Met + Lf group was the lowest among the treatment groups and exhibited a significant difference between the Met + Lf and the Lf groups (Figure 1A). Waist circumference and Lee’s index were obviously higher in the HFD group than in the control group. However, the waist circumference was significantly lower in the Met and Met + Lf groups than in the HFD group (Figure 1B). In addition to the significantly decreased Lee’s index by the treatments, the Lee’s index in the Met + Lf group significantly decreased by 7.3% and 10.1%, compared with the Met and Lf groups, respectively (Figure 1C).

### 3.2. FBG and OGTT

**Figure 2:**
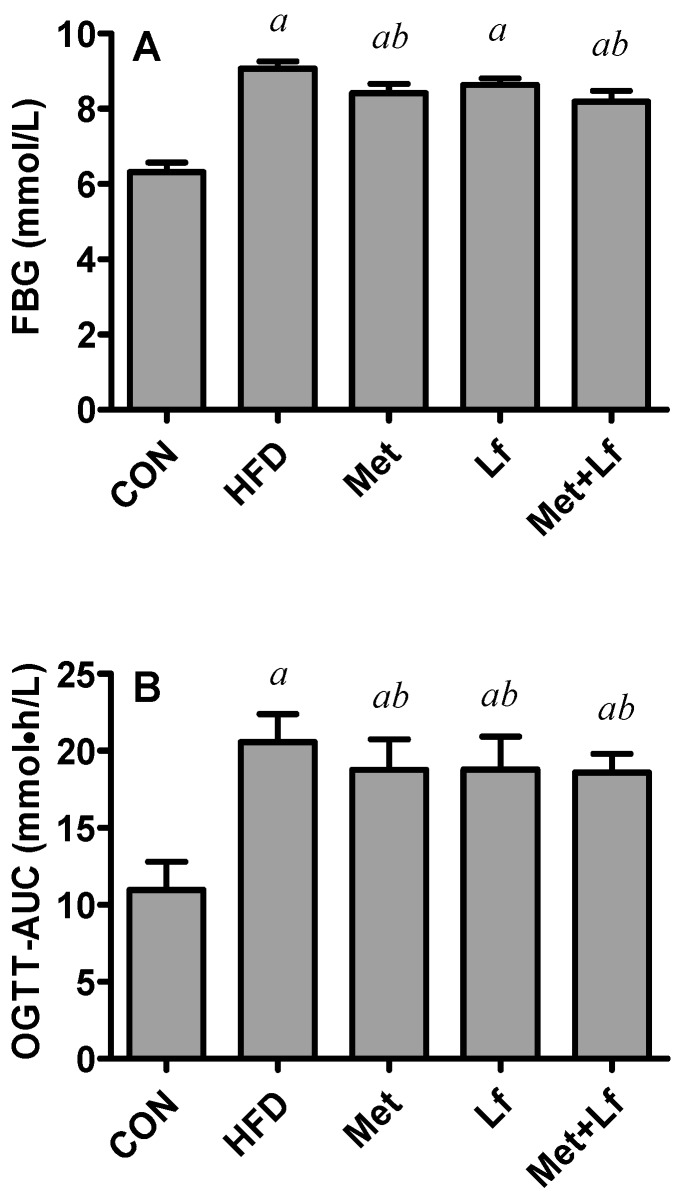
Effect of Met and Lf on FBG (**A**), and OGTT-AUC (**B**) at the end of experiment. Values are express as mean ± SE. ^a^ *p* < 0.05 from CON group; ^b^ *p* < 0.05 from HFD group.

HFD significantly increased FBG levels and treatments with Met, Lf and combination decreased FBG by 7.24%, 4.79%, and 9.64%, respectively, with the significant decrease in the Met and Mer + Lf groups (Figure 2A). OGTT was performed to assess the ability for regulating glucose intake. The values of OGTT-AUC doubled in the HFD group. Compared with HFD group, three treatments significantly decreased OGTT-AUC by approximately 9% (Figure 2B). However, there were no significant differences in FBG or OGTT-AUC among the three treatment groups.

### 3.3. Serum Lipid Profiles, Adipocytokines, and Transaminases

HFD considerably increased the lipid profiles. Compared with the HFD group, the serum levels of TG, TC, and LDL-C significantly decreased in the three treatment groups, and the serum HDL-C level significantly increased in the Met and Met + Lf groups. Furthermore, serum TC level was significantly lower in the Met + Lf group than in the Met and Lf groups, and serum HDL-C level was significantly higher in the Met + Lf group than in the Lf group. For adipocytokine levels, HF significantly increased leptin and decreased adiponectin levels. The three treatments decreased the leptin level to the level of the control group. Compared with the HFD group, serum adiponectin levels were significantly higher in both Met group and Met + Lf group. Furthermore, serum adiponectin level was significantly higher in the Met + Lf group than in the Lf group. No significant difference in serum AST levels were observed among the five groups. Compared with the control group, serum ALT level in the HFD group significantly increased. However, serum ALT levels in the 3 intervention groups were significantly lower than that in the HFD group and restored to the level in the control group (Table 1).

**Figure 3:**
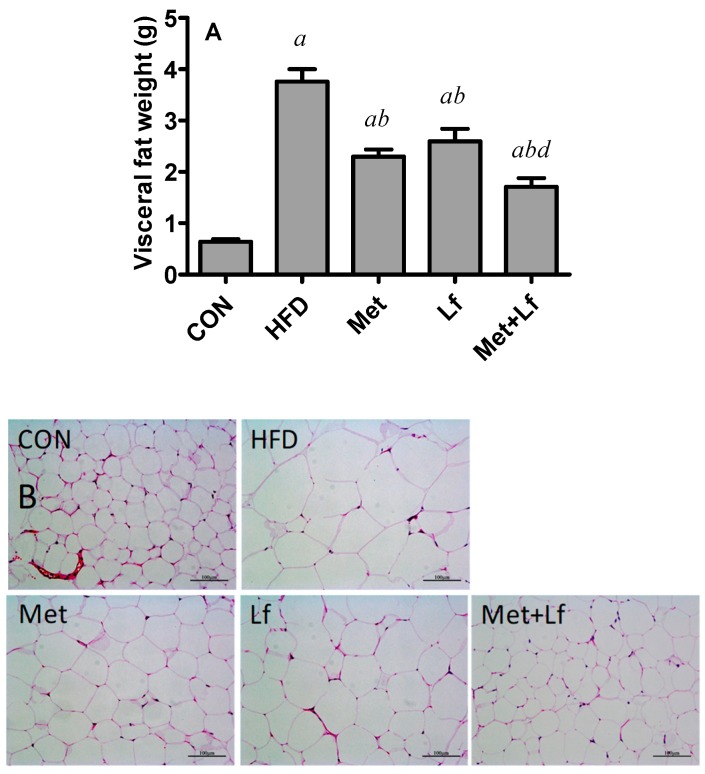

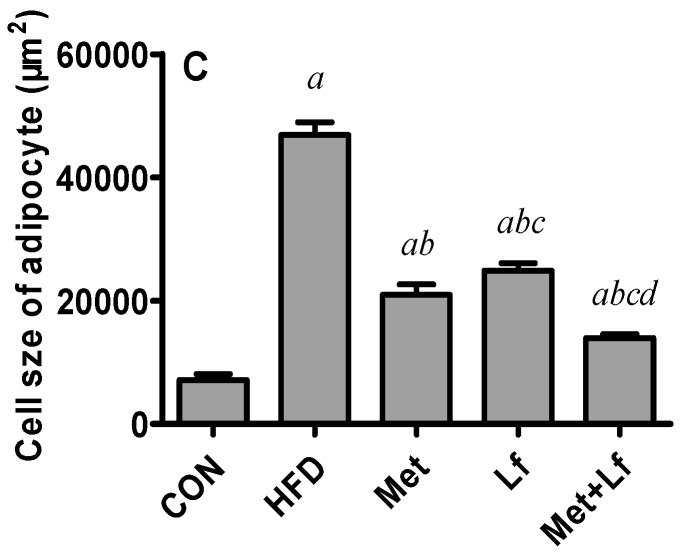
Effect of Met and Lf on visceral fat. (**A**) Visceral fat (excepting mesenteric fat) weight; (**B**) Histological examination of perirenal fat; (**C**) Cell size of adipocyte. Values are express as mean ± SE. ^a^ *p* < 0.05 from CON group; ^b^ *p* < 0.05 from HFD group; ^c^ *p* < 0.05 from Met group; ^d^ *p* < 0.05 from Lf group.

HFD greatly increased the weight of visceral fat (except for mesenteric fat), and its weight was significantly lower in the three treatment groups compared with the HFD group. Fat weight was significantly lower in the Met + Lf group than in the Lf group (Figure 3A). Histological examination showed that HFD enlarged the adipocyte size of perirenal fat, and adipocyte size was markedly reduced by the treatments (Figure 3B). The quantitative analysis was consistent with the histological examination. The adipocyte size in the Met + Lf group significantly decreased by 33.5% and 44.0%, compared with the Met and Lf groups, respectively (Figure 3C).

### 3.4. Hepatic Weight and Lipids

**Figure 4:**
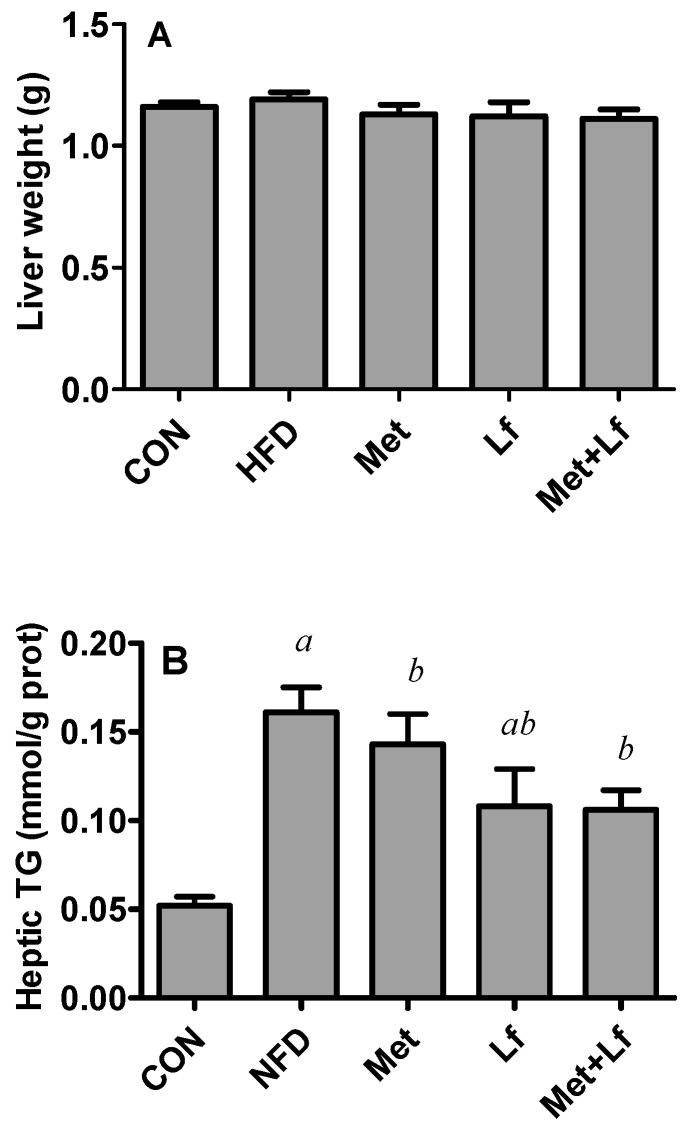

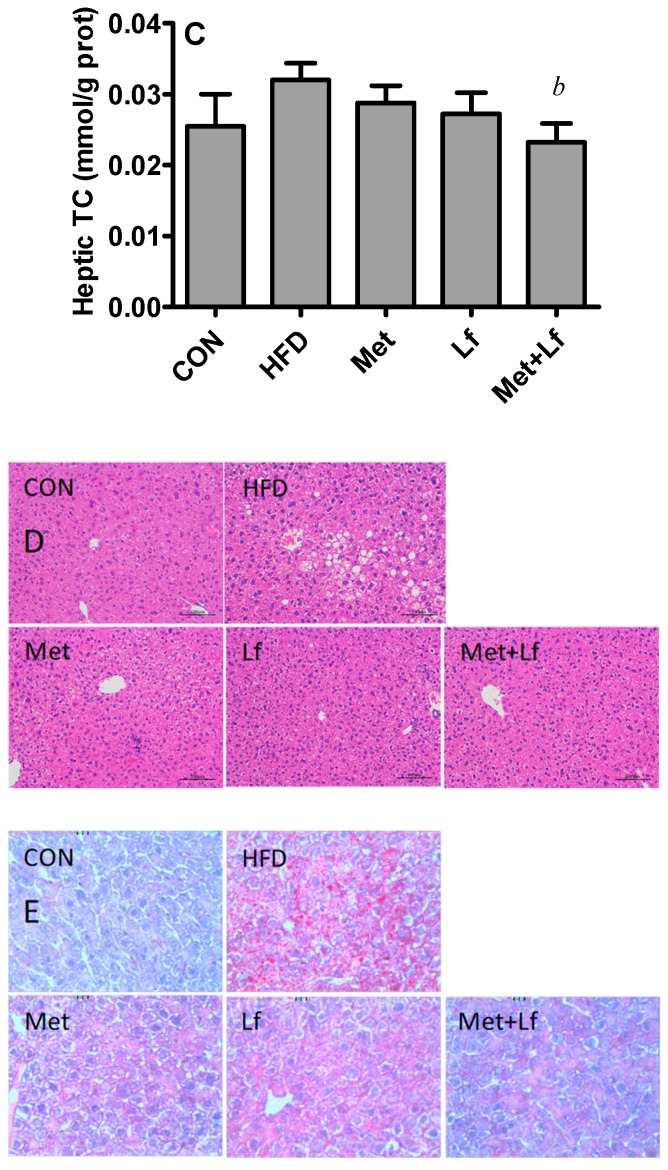
Effect of Met and Lf on hepatic lipid accumulation. (**A**) Liver weight; (**B**) hepatic TG; (**C**) hepatic TC; (**D**) liver tissues stained with HE (200×); and (**E**) Oil Red O (400×). Values are express as mean ± SE. ^a^ *p* < 0.05 from CON group; ^b^ *p* < 0.05 from HFD group.

HFD slightly increased and the treatments slightly decreased hepatic weights without significant differences among the 5 groups (Figure 4A). It should be noted that the treatments significantly suppressed HFD-induced increase of hepatic TG contents (Figure 4B). Compared with the HFD group, the hepatic TC content was significantly decreased in the Lf + Met group, but this was not shown in the Met and Lf groups (Figure 4C). The H and E and Oil Red O stainings of liver tissues showed that HFD caused the hepatic lipids to accumulate. However, hepatic lipid accumulation was alleviated after treatment, and the improvement was more obvious in the Met + Lf group (Figure 4D,E).

### 3.5. Hepatic Protein Expression

**Figure 5:**
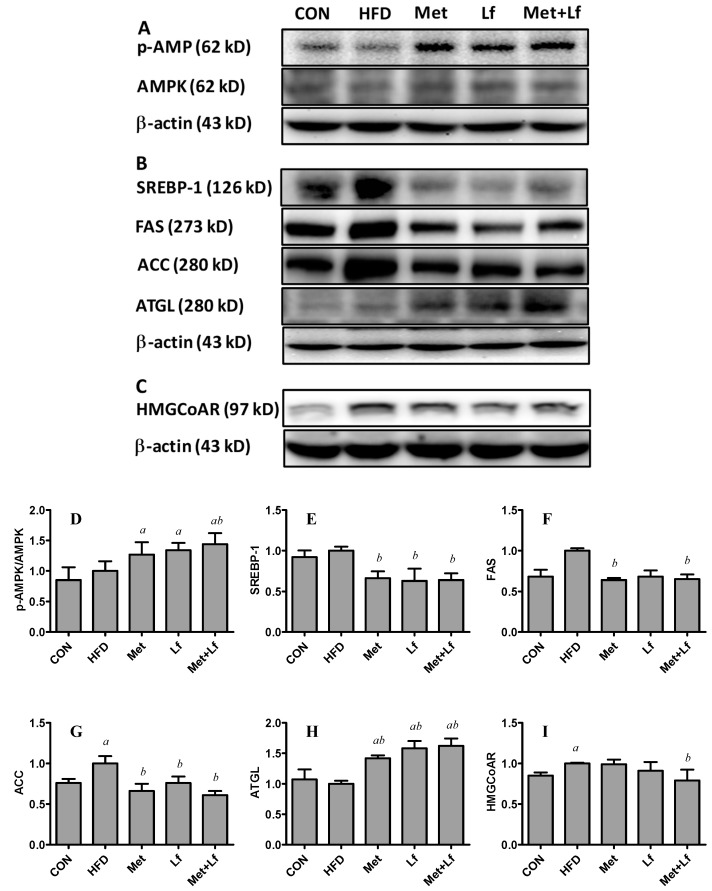
Effect of Met and Lf on the expression of hepatic protein. The protein expression of p-AMPK and AMPK (**A**), SREBP-1, FAS, ACC and ATGL (**B**) and HMGCoAR (**C**) were estimated by Western blotting. Western blots were quantified and the p-AMPK/AMPK ratio (**D**), SREBP-1 (**E**), FAS (**F**), ACC (**G**), ATGL (**H**) and HMGCoAR are shown. Values are express as mean ± SE. ^a^ *p* < 0.05 from CON group; ^b^ *p* < 0.05 from HFD group.

We measured the protein expression related to hepatic lipid metabolism. HFD did not affect the expression of SEREBP-1, FAS, ATGL, and p-AMPK/AMPK; but increased the expression of ACC and HMGCoAR. Although the protein levels of AMPK were unchanged in all groups, the treatments upregulated the p-AMPK protein expression. Thus, the p-AMPK/AMPK ratio was significantly higher in the Met + Lf group than in the HFD group (Figure 5A,D). Compared with the HFD group, the treatments significantly downregulated the protein expression of lipogenic enzymes (FAS and ACC) and SREBP-1 (Figure 5B,E–G), which regulates ACC and FAS [27]. In addition, the treatments significantly upregulated the protein expression of lipolytic enzyme such as ATGL (Figure 5B,H). The protein expression of HMGCoAR was only significantly lower in Met + Lf group than in the HFD group (Figure 5C,I).

## 4. Discussion

In the present study, the mice fed with HFD showed increased body and visceral fat weights. The mice in the HFD group exhibited increased plasma lipids, ALT levels, and hepatic TG content. This suggested that HFD resulted in obesity and dyslipidemia in mice.

Treatment of Met and Lf significantly decreased body weight, waist circumference, Lee’s index, and visceral fat, but had no effect on liver weight. In Li’s study and Woo’s study, the mice were fed with HFD for 12 weeks and Met were treated from the last four weeks. The former study showed a decrease in body weight, epididymal white adipose, and serum TG level with no effect on liver weight [7]; however, the latter study found a decrease in liver weight, with no effects on body weight and adiposity [8]. For Lf, both Sun and Xiong found that long-term administration decreased body weight, fat weight, and serum and hepatic TG in the obese mice maintained on a HFD [18,19]. Similar with our result, the alleviation of hepatic lipid accumulation by Met and Lf treatments was also observed in the H and E and Oil Red O stainings in these animal studies [7,8,12,18]. In the in vitro study, high glucose and insulin increased TG accumulation in the HepG2 cells, whereas Met treatment substantially decreased its accumulation [28]. Lipid droplets increase during differentiation of 3T3-L1 cells, and Lf administration led to a dose-dependent reduction in lipid accumulation [29]. In general, our results were consistent with those obtained from previous studies with different study designs.

Dysregulation of lipid metabolism generally leads to disruption of glucose metabolism. The action of Met, a common oral antihyperglycemic drug to improve glucose tolerance in patients with type 2 diabetes mellitus, has been well understood [8]. Several studies observed a beneficial effect of Lf on glucose homeostasis. Lf supplementation for 15 weeks significantly reduced serum levels of insulin and glucose, and reduced insulin resistance in HFD-induced obese mice [18]. Lf supplementation for eight weeks also decreased fasting and postprandial blood glucose and glucose AUC in obese prone rats feeding with HFD [30]. Although Met and Lf decreased FBG levels and OGTT-AUC, we did not observe additional benefits in the combination group. The synergistical action of Met and Lf for regulating glucose intake needs further studies. Circulating adipocytokines play important roles in glucose and lipid metabolisms [31]. Obese subjects are known to have increased serum leptin and decreased adiponectin levels [32], which is evidenced by our animal study. We found that the treatments decreased leptin and increased adiponectin levels. Meta-analyses demonstrated that Met increased serum adiponectin levels [33], but had no effect on blood leptin [34] when treating type 2 diabetes mellitus. On the other hand, Xiong et al. found that Lf significantly reduced leptin and monocyte chemotatic protein-1 levels but had no effect on adiponectin [19].

Our results were supported by human trials. Met treatment for six months led to higher loss of body weight in non-diabetic obese individuals [4]. Lf consumption for eight weeks decreased body weight, body mass index, waist circumference, and abdominal fat areas in abdominally obese adults [20]. In addition, the patients with non-alcoholic steatohepatitis administrated Met for 48 weeks increased the proportion of patients with normal ALT levels; however, treatment did not affect the proportion of patients with normal AST levels [35]. This result was supported by our animal study.

In the present study, we found that combined treatment exhibited lower body weight, fat weight, serum and hepatic TG levels, suggesting that Met and Lf may act synergistically to improve lipid metabolism. There were no other study to observe the synergistical effects of Met and Lf. In Yang’s study, the rats were divided into control, Met, and sitagliptin groups. Each group was further divided into 4 groups, including one group receiving 20% whey protein, which is the main source of Lf. They found that the rats in Met treatment receiving whey protein had more decreased TG level compared with Met alone [36]. Leucine, which is a branched-chain amino acid, is rich in milk-derived protein. The other indirect evidence was that Met treatment with leucine further reduced hyperlipidemia and hepatic lipid accumulation in diet-induced obese mice [12]. 

The liver is the center of lipid metabolism. The activity of AMPK can be inhibited by obesity, hyperlipidemia and diabetes [21]. Met and Lf are well-known agonists of AMPK [26,27]. Xiong et al. did not found the significant difference in pThr172AMPK/AMPK among the control group, HFD group and Lf+HFD group [19]. In the present study, although AMPK levels were not significantly changed in all groups, Met and Lf phosphorylated AMPK protein. SREBP-1, a key transcription factor in the regulation of lipid synthesis in liver, is downstream of AMPK [37]. Similar to our results, the mRNA and protein expression of SREBP-1c, a main isoform in hepatocytes, was substantially reduced after Met treatment in HepG2 cells [28]. The mRNA level of SREBP-1 was also downregulated by Lf supplementation in HFD-induced obese mice [19]. Lipid metabolism is a complicated process that involves many enzymes. FAS and ACC are the key enzymes for fatty acid synthesis, and ATGL is the main enzyme for triglyceride degradation [27]. These enzymes and SREBP-1 are all controlled by AMPK. As expected, our study found that Met and Lf treatments inhibited the protein expression of FAS and ACC and elevated the protein expression of ATGL. Therefore, Met and Lf decrease lipogenesis and increase lipolysis in the liver to improve lipid metabolism. 

We did not find the obvious difference of the related protein expression between the treatments of alone and combination; however, combination treatment showed more effective on body and fat weights. Furthermore, we did not find the expression change of p-AMPK/AMPK by HFD, suggesting that other signaling pathways may exist in this complex regulation. HMGCoA reductase is an important rate limiting enzyme in cholesterol biosynthesis [22]. Our study found that the combination, but not Met or Lf alone, significantly inhibited its protein expression, which was consistent with the hepatic TC change. Sirt1, which is activated through AMPK-mediated induction of nicotinamide phosphoribosyltransferase, is crucial to the regulatory network for metabolic homeostasis [38]. Banerjee et al. found that the Met–leucine combination stimulated insulin signaling pathway by activating the AMPK/Sirt1 pathway and increased fatty acid oxidation, which was prevented by AMPK or Sirt inhibition [39]. Adipose tissue is categorically divided into white adipose tissue which is characterized by lipid storage, and brown adipose tissue for whose function is energy dissipation. In an animal study, the expression levels of white adipose tissue-associated genes significantly reduced, and the expression levels of brown adipose tissue-associated genes increased in the livers when obese mice were orally administered with Met for 14 weeks [40]. Lf stimulation also promoted UCP1 gene expression in brown adipocytes, leading to increased energy expenditure in human reprogrammed brown adipocytes [41]. In addition, animal studies demonstrated that Lf administration modulated gut microbiota [18], and appetite regulatory pathways in the brain and gut-derived signals [30], which was possible in favor of Met actions.

In conclusion, we found that Met and Lf, either alone or in combination, improved HFD-induced obesity and lipid metabolism. The actions of Met and Lf were partially due to the AMPK-related pathway. However, further studies are needed to elucidate the molecular mechanisms underlying the effect of Met treatment with Lf on the regulation of lipid metabolism.

## Figures and Tables

**Table 1:** The levels of serum lipid profiles and transaminases at the end of the experiment.

	TG (mmol/L)	TC (mmol/L)	HDL (mmol/L)	LDL (mmol/L)	Leptin (ng/mL)	Adiponectin (ng/mL)	AST (U/L)	ALT (U/L)
CON	1.55 ± 0.1	3.57 ± 0.41	1.51 ± 0.07	0.16 ± 0.02	2.21 ± 0.16	8.97 ± 0.26	152.94 ± 9.61	37.04 ± 2.39
HFD	1.78 ± 0.06 ^a^	8.89 ± 0.72 ^a^	2.85 ± 0.17 ^a^	0.29 ± 0.01 ^a^	7.48 ± 0.96 ^a^	7.79 ± 0.36 ^a^	156.83 ± 13.25	44.47 ± 2.39 ^a^
Met	1.51 ± 0.06 ^b^	7.41 ± 0.51 ^ab^	3.29 ± 0.06 ^ab^	0.23 ± 0.01 ^b^	2.13 ± 0.27 ^b^	9.15 ± 0.41 ^b^	144.57 ± 5.40	33.89 ± 1.35 ^b^
Lf	1.4 ± 0.04 ^b^	6.81 ± 0.52 ^ab^	2.9 ± 0.08 ^ac^	0.24 ± 0.01 ^a^	2.64 ± 0.37 ^b^	8.11 ± 0.33 ^c^	150.46 ± 5.39	34.28 ± 1.71 ^b^
Met + Lf	1.41 ± 0.04 ^b^	5.64 ± 0.19 ^abcd^	3.27 ± 0.08 ^abd^	0.24 ± 0.01 ^ab^	1.95 ± 0.13 ^b^	9.61 ± 0.48 ^bd^	138.59 ± 7.78	33.26 ± 2.1 ^b^

Values are express as mean ± SE. ^a^ *p* < 0.05 from CON group; ^b^ *p* < 0.05 from HFD group; ^c^ *p* < 0.05 from Met group; ^d^ *p* < 0.05 from Lf group.3.4. Visceral fat weight and adipocyte size; TG: triglyceride; TC: total cholesterol; HDL: high density lipoprotein; LDL: low density lipoprotein; AST: aspartate transaminases; ALT: alanine transaminase; CON: control group; HFD: high-fat diet; Met: Metformin; Lf: lactoferrin.
